# Genome-wide analysis of *EgEVE_1*, a transcriptionally active endogenous viral element associated to small RNAs in *Eucalyptus* genomes

**DOI:** 10.1590/1678-4685-GMB-2016-0086

**Published:** 2017-02-23

**Authors:** Helena Sanches Marcon, Juliana Costa-Silva, Alan Péricles Rodrigues Lorenzetti, Celso Luis Marino, Douglas Silva Domingues

**Affiliations:** 1 Departamento de Botânica, Instituto de Biociências, Universidade Estadual Paulista “Júlio de Mesquita Filho” (UNESP), Rio Claro, SP, Brazil.; 2 Departamento de Genética, Instituto de Biociências, Universidade Estadual Paulista “Júlio de Mesquita Filho” (UNESP), Botucatu, SP, Brazil.; 3 Programa de Pós-graduação em Bioinformática, PPGBIOINFO, Universidade Tecnológica Federal do Paraná, Cornélio Procópio, PR, Brazil.; 4 Programa de Pós-graduação em Genética e Biologia Molecular, Universidade Estadual de Londrina, Londrina, PR, Brazil.

**Keywords:** Pararetrovirus, horizontal transfer, Eucalyptus, Caulimovirus, insertion

## Abstract

Endogenous viral elements (EVEs) are the result of heritable horizontal gene transfer from viruses to hosts. In the last years, several EVE integration events were reported in plants by the exponential availability of sequenced genomes. *Eucalyptus grandis* is a forest tree species with a sequenced genome that is poorly studied in terms of evolution and mobile genetic elements composition. Here we report the characterization of *E. grandis* endogenous viral element 1 (*EgEVE_1*), a transcriptionally active EVE with a size of 5,664 bp. Phylogenetic analysis and genomic distribution demonstrated that *EgEVE_1* is a newly described member of the Caulimoviridae family, distinct from the recently characterized plant Florendoviruses. Genomic distribution of *EgEVE_1* and Florendovirus is also distinct. *EgEVE_1* qPCR quantification in *Eucalyptus urophylla* suggests that this genome has more *EgEVE_1* copies than *E. grandis. EgEVE_1* transcriptional activity was demonstrated by RT-qPCR in five *Eucalyptus* species and one intrageneric hybrid. We also identified that *Eucalyptus* EVEs can generate small RNAs (sRNAs),that might be involved in *de novo* DNA methylation and virus resistance. Our data suggest that EVE families in *Eucalyptus* have distinct properties, and we provide the first comparative analysis of EVEs in *Eucalyptus* genomes.

## Introduction

In the last years, the burst of plant genome sequences has uncovered innumerable cases of horizontal gene transfer (HGT). HGT is the DNA flow between unrelated species. For many years, HGT events were considered rare and uncommon, but numerous genome analyses have since revealed the wide extent of HGT in plants (Richardson and Palmer, 2007; Yue *et al.*, 2012). Viruses play important roles in HGT, once many studies detected viral sequences integrated into several plant genomes (Bertsch *et al.*, 2009; Geering *et al.*, 2014; Fonseca *et al.*, 2016). These viral DNA sequences present within the genomes of non-viral organisms are known as Endogenous Viral Elements (EVEs; Holmes, 2011). EVEs can consist of an entire viral genome or only a partial fragment (Chu *et al.*, 2014). The function of EVEs remains unclear, but some studies suggest a relationship between viral fragments in genomes and antiviral immunity (Aswad and Katzourakis 2013; Fonseca *et al.*, 2016). Genomic EVE regions can also act as generators of several types of virus-derived small RNAs (sRNAs; Sharma *et al.*, 2013) in some plant species (Becher *et al.*, 2014; Fonseca *et al.*, 2016). The most abundant virus integrations in plants are from Caulimoviridae, a Pararetrovirus family. Using comparative genomics approaches, Caulimovirus-related sequences were identified in several angiosperms (Chabannes and Iskra-Caruana, 2013), including *Eucalyptus grandis*, and they comprise a significant fraction of these plant genomes (Geering *et al.*, 2014).

Previous works have already reported the serendipitous discovery of EVEs in plants during large-scale annotation of LTR retrotransposons (LTR-RTs) (Piednoël *et al.*, 2013) or during next generation sequencing analyses of genomes and transcriptomes (Villacreses *et al.*, 2015; Fonseca *et al.*, 2016). A similar case happened during the annotation of transcriptionally active LTR-RTs in *Eucalyptus* (Marcon *et al.*, 2015). An *E. camaldulensis* EST (GenBank accession FY783514), firstly identified because it contains a reverse transcriptase sequence, was in fact the fragment of a *Caulimovirus*. Alignment analysis of this sequence in the *E. grandis* genome (Myburg *et al.*, 2014) led us to the identification of a new EVE family in this genus. In this study, using publicly available genomic and transcriptomic *E. grandis* resources, we report the molecular characterization of this new EVE family, named *E. grandis* endogenous viral element 1 (*EgEVE_1*). We extended *in silico* analyses of *EgEVE_1,* carrying out comparative quantitative copy number analyses in two *Eucalyptus* species and performing transcriptional analysis in five *Eucalyptus* species and one intrageneric hybrid. We also compared *EgEVE_1* to the Caulimoviridae genus called ‘Florendovirus’, recently identified in the *E. grandis* genome (Geering *et al.*, 2014), in terms of phylogenetic position, genomic distribution and the capacity of generating sRNAs.

This study is the first fine-scale analysis of EVEs in *Eucalyptus* and an important step in the molecular characterization of mobile genetic elements in this woody plant genus.

## Material and Methods

### Virus-like sequences in *Eucalyptus grandis* genome

During the characterization of transcriptionally active LTR-RTs in the *Eucalyptus* genus (Marcon *et al.*, 2015), we found a reverse transcriptase fragment in an *E. camaldulensis* EST sequence (GenBank accession FY783514). Similar to Piednoël *et al.* (2013), after careful checking using CENSOR implemented in RepBase (Kohany *et al.*, 2006), we discovered that this reverse transcriptase is in fact a partial sequence from a *Caulimovirus*.

Using the reverse transcriptase sequence of *E. camaldulensis* EST as a query, we identified a genomic region with high similarity (85% in BLASTN) in *E. grandis* genome scaffold 7. After manual checking of this hit using CENSOR and Repbase, we defined position 10,999,785 to 11,005,448 as a reference for further analyses. For a comparative analysis, we included four consensus *Florendovirus* sequences recently identified in *E. grandis* genome (Geering *et al.*, 2014).

Conserved domains were identified using the CDD tool from NCBI (http://www.ncbi.nlm.nih.gov/Structure/cdd/wrpsb.cgi) and were manually inspected.

### Comparative analyses and endogenous viral family name assignment

To verify the relationships among EVEs, reverse transcriptase (RVT) regions were used to build a phylogenetic tree. RVT sequences for this analysis were the same as the ones used in a previous analysis of EVE sequences in the “Maqui Berry” genome (Villacreses *et al.*, 2015). Nucleotide sequences were aligned using MUSCLE (Edgar, 2004) with default parameters, and the phylogenetic trees were generated using MEGA 7.0 (Kumar *et al.*, 2016), applying the Maximum Likelihood method, with 1,000 bootstrap replicates. After performing a model test in MEGA, the General Time Reversible substitution model with Gamma distributed Invariant sites (GTR+I) was used. Gap positions were excluded when present in more than 5% of the sequences.

The new EVE found in *E. grandis* formed a novel lineage within the Caulimoviridae family and was, thus, named *EgEVE1* (*E. grandis* Endogenous virus element 1). The Florendoviruses previously identified in *E. grandis* genome were named as *EgFLOR* (Florendovirus from *E. grandis*) 1 to 4.

### Copy number determination in *E. grandis* genome and diversity analysis

The copy number of *EgEVE1* and of four Florendovirus families found in *E. grandis* genome was determined using MEGABLAST similar to Marcon *et al.* (2015), using the 2.0 genome version deposited at Phytozome (https://phytozome.jgi.doe.gov/pz/portal.html). For copy number estimation, we considered only the ones that covered over 80% of the query and with nucleotide similarity over 80% after manual inspection.

The average divergence (Pi) in RVTs among *EgEVE* members was calculated using DnaSp program (Librado and Rozas 2009).

### *Eucalyptus* spp EST screening

For an initial evaluation of the transcriptional activity of *EgEVE1* and *EgFLOR1-4*, reference sequences were used as BLASTN queries against *Eucalyptus* ESTs from the EUCANEXT database (Nascimento *et al.*, 2011; Salazar *et al.*, 2013; http://bioinfo03.ibi.unicamp.br/eucalyptusdb/), in a approach similar to that of Marcon *et al.* (2015).

### *EgEVE1* relative quantification and transcriptional analysis

A comparative quantification by qPCR of *EgEVE1* reverse transcriptase was performed between the *E. grandis* and *E. urophylla* genomes, using a single-copy gene (DUR3) as a reference. Primers for *EgEVE1* quantification were: EgEVE_RVT_F 5’-CCAAGATGATAAGTTCCC TTTACC-3’ and EgEVE_RVT_R 5’-GGTGGAATTTG GAATAGATGTGG-3’. We followed the same procedures used in a previous study from our group (Marcon *et al.*, 2015). We also evaluated the transcriptional activity of *EgEVE1* reverse transcriptase in *E. grandis, E. brassiana, E. saligna, E. tereticornis, E. urophylla* and in one hybrid *E. grandis* x *E. urophylla* (termed “E. urograndis”). RT-qPCR was used to identify transcriptional activity in leaves, stalks and secondary roots, in physiological conditions and under osmotic stress. Overall procedures for the qPCR assays, including normalization, and plant harvesting, were the same as those described in Marcon *et al.* (2015).

RT-qPCR efficiency was calculated using Linreg v. 2013.0 (Ruijter *et al.*, 2009), and reactions with efficiency > 90% were considered for further analysis. Relative expression was calculated using the ΔΔCt method (Livak and Schmittgen, 2001) with the formula (1 + E)^ΔΔCt^, where E represents the efficiency. Statistical analysis was performed using Assistat 7.7 beta (Silva and Azevedo, 2009). We used one-way analysis of variance (ANOVA), and in cases where significant differences were found, the Least Square Deviation (LSD) method for multiple comparisons was applied. Results were considered significant at P < 0.05. The tissue or organ with the lowest expression (highest Ct) was used as calibrator (expression value = 1).

### Small RNA mapping analysis

Public data from *E. grandis* small RNA sequencing (Levy *et al.*, 2014, NCBI accession GSE58367) was used to map small RNAs against virus-like sequences to check if they may be regulated by post-transcriptional pathways. This is the only publicly available sRNA sequencing data for this genus, comprising 6,891,830 valid reads, obtained from 14-day sterile seedlings.

Raw read quality was assessed using FastQC version 0.10.1 (http://www.bioinformatics.babraham.ac.uk/projects/fastqc/). Trimmomatic version 0.35 (Bolger *et al.*, 2014) was used to preprocess raw reads. In summary, sequencing adapters, overrepresented sequences and reads < 16nt or > 28nt were removed. Only reads with average phred quality > 30 were maintained. Using the FASTQ/A Collapser tool from FASTX-Toolkit (http://hannonlab.cshl.edu/fastx-toolkit), we obtained non-redundant small RNA sequences. This set was filtered using Bowtie 2 (Langmead and Salzberg, 2012) under stringent parameters to remove sequences from chloroplasts, mitochondria, tRNAs, rRNAs and snoRNAs. Non-reduntant sequences were mapped against *E. grandis* chloroplast (GenBank Accession NC_014570) and ribosomal units obtained in the SILVA ribosomal RNA gene database (Quast *et al.*, 2013), *Gossypium barbadense, Solanum lycopersicum* and *Vitis vinifera* mitochondria (GenBank accessions AFYB00000000, NC_028254 and NC_012119), *Populus tricocharpa* and *Vitis vinifera* tRNAs from GtRNAdb (Chan and Lowe, 2009) and snoRNAs from the Plant snoRNA database (http://bioinf.scri.sari.ac.uk/cgi-bin/plant_snorna/home). Sequences that matched these references were discarded for further analysis. After this filtering, a total of 615,801 non-redundant sequences were mapped against *EgEVE1* and *EgFLOR1-4* sequences under stringent parameters, with no gaps and mismatches, using Bowtie2.

## Results

### Identification of a novel EVE in *Eucalyptus grandis* genome

We identified a sequence highly similar to an endogenous caulimovirus in the *E. grandis* genome using an *E. camaldulensis* EST as query. After manual annotation of a reference region (scaffold7: 10999785 to 11005448), we determined an EVE region of 5,664 nucleotides (nt), named *EgEVE_1*.

*EgEVE_1* has three retroviral domains: a reverse transcriptase (RVT- cd01647) with 539 nt, a ribonuclease H (RNase H - cd09274) with 362 nt, and a pepsin-like aspartate protease (PEP - cd00303) with 245 nt (Table 1). All domains were in the same reading frame of the sense strand.

**Table 1:** *EgEVE_1* domains and related gene products.

ORF identification	Start Position	End Position	Length (bp)
RVT^1^	2756	3295	539
RNase H^2^	3572	3934	362
PEP^3^	2186	2431	245

1 reverse transcriptase;

2 ribonuclease H;

3 pepsin-like aspartate protease

### Comparative phylogenetic structure shows that *EgEVE_1* is not related to Florendoviruses

**Figure 1:**
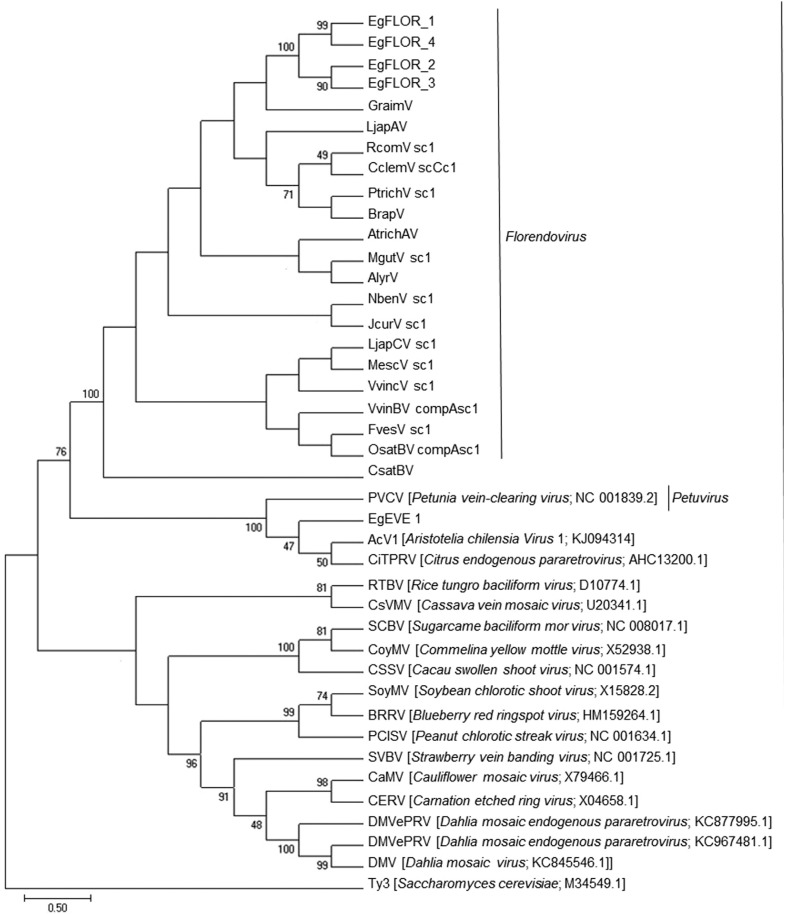
Phylogenetic analysis of reverse transcriptase domain from the Caulimoviridae family, endogenous pararetroviruses. *Ty3* retrotransposon was used as an outgroup.

We classified *EgEVE_1* among viral families using the RVT sequences from previous studies on plant EVEs (Geering *et al.*, 2014; Villacreses *et al.*, 2015) (Figure 1). *EgEVE_1* is highly related to the Caulimoviridae family, especially with Petuvirus, clustering with *PVCV* and *AcV1* (Villacreses *et al.*, 2015). This position suggests that *EgEVE_1* may be considered part of the same genus (Figure 1), although a more comprehensive characterization using related virus sequences and experimental assays are needed to better corroborate this hypothesis. More importantly, we could demonstrate that *E. grandis* Florendoviruses (*EgFLOR*1-4, Figure 1, Figure S1), the sole *Eucalyptus* EVEs identified up to date, belong to another clade. In this way we confirmed that *EgEVE_1* is a new family of pararetroviruses identified in the *E. grandis* genome.

To further confirm that *EgEVE_1* belongs to a new family, we identified complete sequences in *E. grandis* genome.

### *EgEVE_1* distribution in the *E. grandis* genome: a comparative analysis with Florendoviruses

We identified six copies of *EgEVE_1* in the *E. grandis* genome on four *E. grandis* chromosomes. Copy numbers for *EgFLOR* families ranged from 2 to 26 (Table 2), reinforcing that they belong to another group of EVEs. Among Florendoviruses, *EgFLOR_1* has the highest copy number, while *EgFLOR_3* has only two copies (Table 2). In Table S1 (supplementary material) we detail coordinates of each complete copy for the *EgEVE* and *EgFLOR* families.

**Table 2:** Genomic distribution and diversity of EVE families.

EVE family	Copy Number	Chromosomes	Diversity (Pi)
*EgEVE_1*	6	7, 9, 10, 11	0.37 ± 0.0092
*EgFLOR_1*	26	1, 2, 3, 4, 5, 6, 7, 8, 9, 11	0.27 ± 0.0001
*EgFLOR_2*	13	1, 2, 3, 4, 5, 6, 7, 8, 9, 11	0.28 ± 0.0009
*EgFLOR_3*	2	5, 7	0.12 ± 0.25
*EgFLOR_4*	23	1, 2, 3, 4, 5, 6, 7, 8, 9, 11	0.28 ± 0.0001

The diversity (Pi) of *EgEVE_1* complete sequences was higher than the one observed for *EgFLOR* families (Table 2), reinforcing that they have dissimilar genomic features.

### Comparative genomic quantification between *E. grandis* and *E. urophylla*

**Figure 2:**
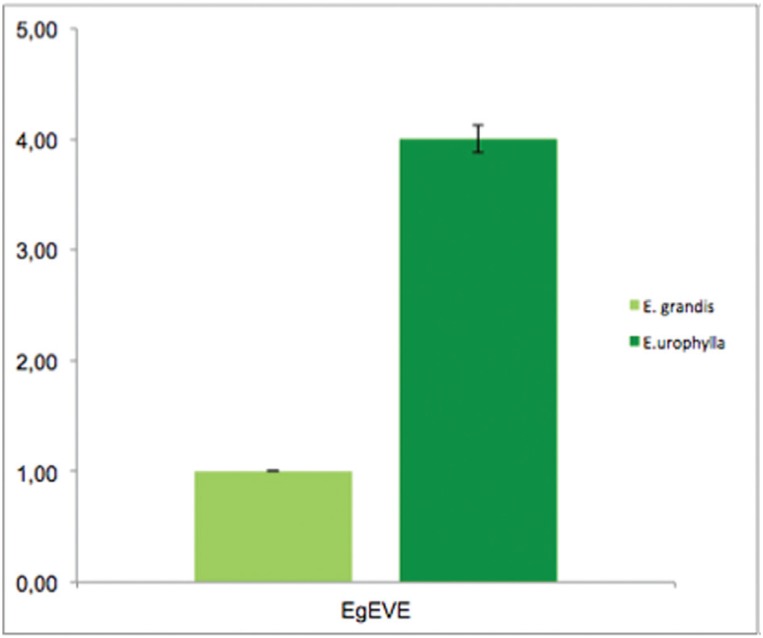
*EgEVE_1* RVT copies from *E. grandis* and *E. urophylla* using qPCR.

Since we were able to find EVEs in *E. grandis* using a congeneric species sequence as a query, we hypothesized that a qPCR analysis in a conserved region of *EgEVE_1* would allow a comparative quantification of this EVE family among *Eucalyptus* species. In this way, having in mind that *in silico* analyses were based on the *E. grandis* genome, we used this species to run a comparative quantification of *EgEVE_1* RVT domain by qPCR in *E. urophylla,* similar to the one performed by Marcon *et al.* (2015), using a single-copy gene as a reference. Our analyses suggest that *E. urophylla* could have more *EgEVE_1* copies than *E. grandis* (Figure 2).

### *EgEVE_1* transcriptional activity in *Eucalyptus* species and in different organs

This is the first report on transcriptional activity of EVEs in forest trees. We BLAST searched the transcriptome of six *Eucalyptus* species deposited in the EUCANEXT database (Nascimento *et al.*, 2011; Salazar *et al.*, 2013), using *EgEVE_1* and *EgFLOR1-4* as queries. We did not find any hit for *EgFLOR1-4*, indicating that Florendoviruses are not transcriptionally active in *Eucalyptus* genomes. *EgEVE_1* only showed similarity with ESTs from *E. calmadulensis* (Supplementary material Table S2).

**Figure 3:**
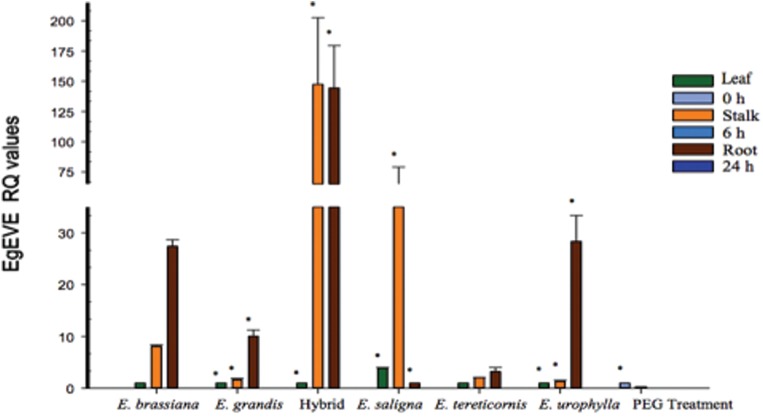
Transcriptional profile of *EgEVE* in three tissues from five *Eucalyptus* species and one interspecific hybrid using RT-qPCR. Asterisk indicates differential expression (*p ≤ 0.05, ANOVA followed by LSD test).

We also analyzed *EgEVE_1* transcriptional levels using RT-qPCR for three tissues (leaves, stalk and secondary roots) from five *Eucalyptus* species (*E. brassiana, E. grandis, E. saligna, E. tereticornis* and *E. urophylla*) and one intrageneric hybrid (*E. grandis* x *E. urophylla* – termed “E. urograndis” to facilitate discussion). We also evaluated secondary roots from *E. grandis* under osmotic stress imposed by PEG treatment (Rodrigues *et al.*, 2013) (Figure 3).

The highest transcriptional levels for *EgEVE_1* were found in stalks and roots from E. urograndis and *E. saligna* (Figure 3). Interestingly, *EgEVE_1* displayed low transcriptional activity in leaves (Figure 3). Considering that most transcriptome analyses use leaves, this may explain the lack of *EgFLOR* in expressed sequences. *EgEVE_1* transcriptional levels were repressed in roots submitted to osmotic stress by PEG treatment (Figure 3).

### *Eucalyptus* EVEs as sources of small RNAs

There is evidence that EVEs might act as sources of sRNAs, probably shaping epigenic features and/or having a role on antiviral defenses (Becher *et al.*, 2014; Geering *et al.*, 2014; Fonseca *et al.*, 2016). To check if *Eucalyptus* EVEs could be involved in sRNA production, we mapped filtered non-redundant sRNAs ranging from 16 to 26 nt with zero mismatches to consensus EVE sequences (*EgEVE_1* and *EgFLOR1-4*). Although the numbers of sRNAs matches are probably underestimated due to polymorphisms between reference copies and genomic sequences, this analysis can provide an initial overview of sRNA production in *Eucalyptus* EVEs.

**Figure 4:**
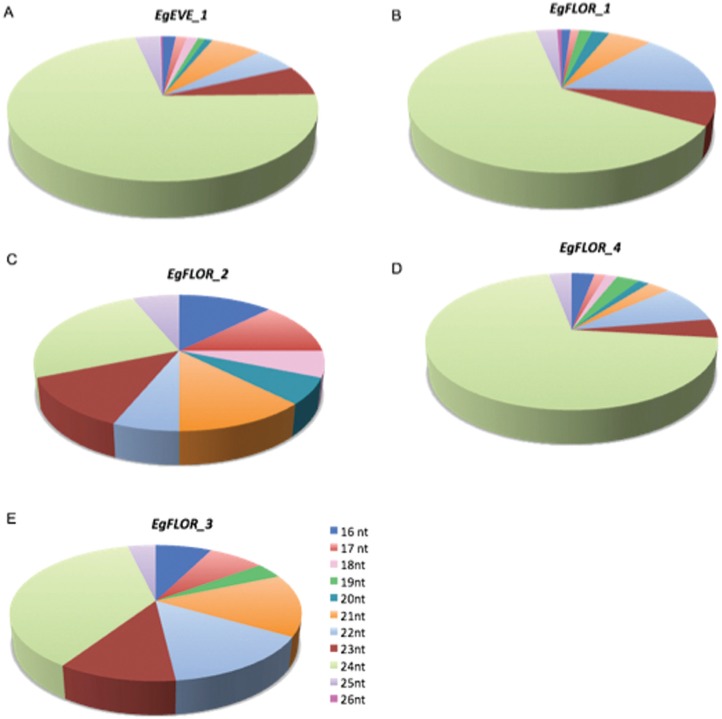
Size variation of sRNAs (16 to 26 nucleotiotides) according to EVE. (A) *EgEVE_1*, (B) *EgFLOR_1*, (C) *EgFLOR_2*, (D) *EgFLOR_3,* (E) *EgFLOR_4.*

We mapped a total of 727 sRNA reads (Figure 4; Table S3). *EgEVE_1* was the element with most mapped reads (434) and *EgFLOR2* had the lowest number of mapped reads (16), and the most abundant class for all EVEs was 24-nt sRNAs (Supplementary material Table S3; Figure 4), a class usually associated to transposable elements and repetitive sequences and involved in RNA-directed DNA methylation (Zhang and Zhu, 2011; Parent *et al.*, 2012). 24 nt sRNAs have already been associated to plant-integrated pararetroviruses (Becher *et al.*, 2014).

**Figure 5:**
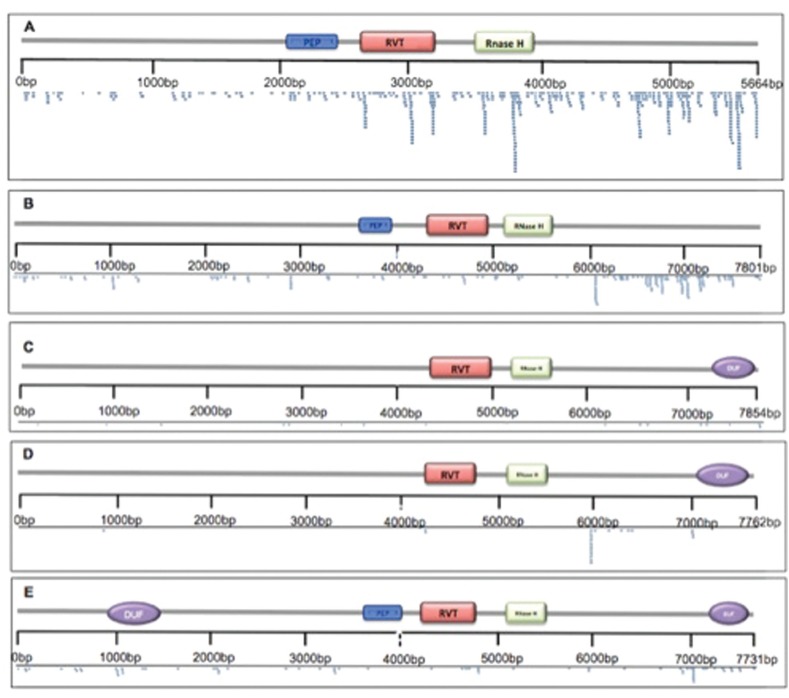
*Eucalyptus grandis* small RNA reads distribution along EVE reference sequences. (A) *EgEVE_1*, (B) *EgFLOR_1*, (C) *EgFLOR_2,* (D) *EgFLOR_3*, (D) *EgFLOR_4.*

In all EVEs, most sRNAs were mapped in the 3’ region. In the case *EgEVE_1*, we observed a clustered mapping of sRNAs in RVT and RNAseH regions (Figure 5), suggesting a prominent role of these regions in sRNA regulation.

## Discussion

Our data report the first transcriptionally active EVE in the *E. grandis* genome, *EgEVE_1*, using bioinformatics and experimental approaches. Similar to EVEs that were recently described in several plant genomes (Chabannes and Iskra-Caruana, 2013; Becher *et al.*, 2014; Villacreses *et al.*, 2015), *EgEVE_1* is also classified as being close to the genus *Petuvirus* within the Caulimoviridae.

We could not recover the *Gag* domain of *EgEVE_1* in any of its genomic copies. Such arrangements of fragmented copies dispersed at several genomic loci have been also described in EVEs from *Musa* and *Nicotiana* species (Chabannes and Iskra-Caruana, 2013). On the other hand, *Eucalyptus* Florendoviruses (*EgFLOR* families) are bigger than *EgEVE_1* (7731 to 7854 bp), with similar size when compared to other pararetroviruses (Calvert *et al.*, 1995; Villacreses *et al.*, 2015).

*EgEVE_1* is clearly distinct from the *EgFLOR* families by phylogenetic (Figure 1) and genomic (Table 1) analysis. Floredoviruses also contain a domain that encodes a putative protein of unknown function (Figure 5).

The quantification of genomic repetitive units by comparative qPCR has been performed in several species (Baruch and Kashkush, 2012; Yaakov *et al.*, 2013; Marcon *et al.*, 2015). The genomes of *E. grandis* (1C = 630 Mb) and *E. urophylla* (1C = 640 Mb) are of similar size and diverged < 20 Mya (Myburg *et al.*, 2014), making them a good congeneric pair for comparative analyses of *EgEVE_1* distribution in the two genomes. *EgEVE_1* showed more copies (approximately four times more) in the *E. urophylla* genome than in *E. grandis*, suggesting recombination and/or recurrent invasion of this EVE family.

The transcriptomic data associated with *EgEVE_1* and *EgFLOR* families gave an initial overall picture of transcriptional activity of these elements in *Eucalyptus* genomes. *EgFLOR* families seem to have a very low transcriptional activity, since we could find transcripts for only one family. Further experimental analyses using other organs should better address the question of whether these elements are in fact “silent” components of *Eucalyptus* genomes.

To our knowledge, *EgEVE_1* is the transcriptionally most active EVE found in a *Eucalyptus* genome up to date. Furthermore, RT-qPCR analyses also showed that *EgEVE_1* has transcriptional activity differences among *Eucalyptus* spp. tissues and species (Figure 3).

The transcriptional activity of *EgEVE_1* suggests that this family can act in small interference RNA (siRNA) pathways mediated by virus infections. This feature has also been observed in RT-qPCR analyses of other pararetroviruses in different plants, which revealed a low level of transcription associated to asymptomatic plants under normal growth conditions (Noreen *et al.*, 2007; Villacreses *et al.*, 2015). In support of this hypothesis, our sRNA analysis showed that the “24 nt pattern” is prevalent in all analyzed of EVEs, which are known to be associated to viral siRNA pathways (Sharma *et al.*, 2013) and *de novo* DNA methylation (Blevins *et al.*, 2015), thus also explaining the observed low transcriptional activity of *EgFLOR* families due to methylation. In this way, EVE sequences integrated in the *Eucalyptus* genome may have roles in both DNA methylation patterns, as well as virus-plant interactions, warranting further studies on the impact of EVEs under biotic stress conditions.

In summary, this first fine-scale analysis of EVE integration in *Eucalyptus* species highlighted the importance of mobile elements in reshaping genomes and providing molecular tools to confer viral resistances in a tree genome.

## Supplementary material

The following online material is available for this article:

Table S1 - Distribution of EVE elements in *Eucalyptus grandis* genome.

Table S2 - Expressed sequence tags (ESTs) matching to EVE elements.

Table S3 - sRNAs mapped to *Eucalyptus* EVEs.

Figure S1 - Phylogenetic analysis of reverse transcriptase domain from *EgEVE* copies.
