# Supplementary material for: Genome-wide analysis of EgEVE_1, a transcriptionally active endogenous viral element associated to small RNAs in Eucalyptus genomes
Source: Genet Mol Biol. 2017 Feb 23;40(1 Suppl 1):217–25. doi: 10.1590/1678-4685-GMB-2016-0086 (PMC5452135; doi:10.1590/1678-4685-GMB-2016-0086)
Supplement: Supplementary file 1 [file 1415-4757-gmb-1678-4685-GMB-2016-0086-Suppl04.pdf]

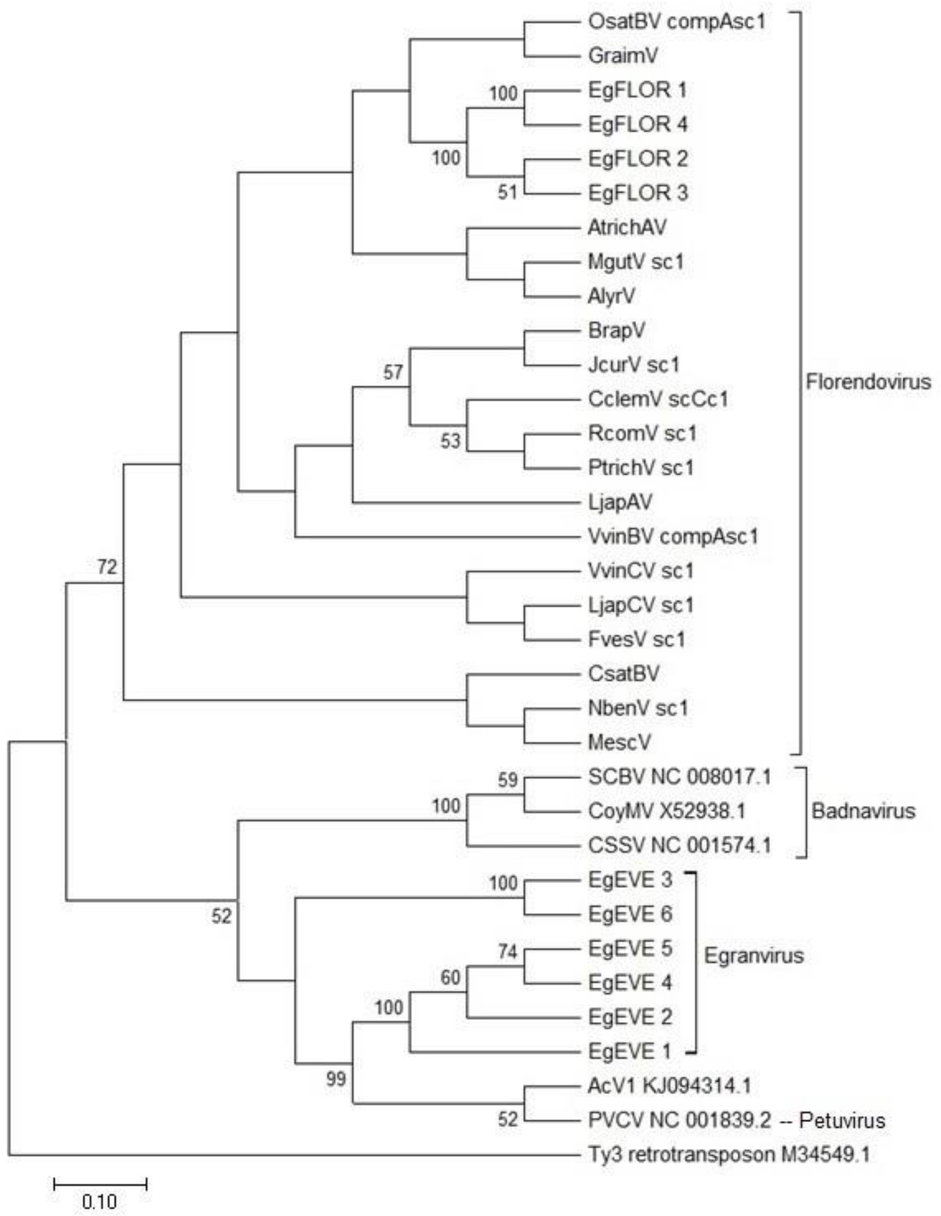

Figure S1 - Phylogenetic analysis of reverse transcriptase domain from *EgEVE* copies, compared to selected Caulimoviridae members. *Ty3* retrotransposon was used as an external group.
