# Supplementary material for: Genome-wide analysis of EgEVE_1, a transcriptionally active endogenous viral element associated to small RNAs in Eucalyptus genomes
Source: Genet Mol Biol. 2017 Feb 23;40(1 Suppl 1):217–25. doi: 10.1590/1678-4685-GMB-2016-0086 (PMC5452135; doi:10.1590/1678-4685-GMB-2016-0086)
Supplement: Supplementary file 2 [file 1415-4757-gmb-1678-4685-GMB-2016-0086-Suppl01.pdf]

Table S1 - Distribution of EVE elements in *Eucalyptus grandis* genome.

EVEs	Chromosome	Start	End
EgEVE_1	7	9066145	9091321
	7	17293165	17318366
	7	10989785	11015448
	9	118818	143958
	11	19166462	19191330
	10	26213692	26238458
EgFLOR_1	7	3126120	3133916
	7	38351411	38359179
	7	43159241	43166382
	7	47515161	47522956
	8	6849737	6857524
	8	36161237	36169008
	8	41731084	41738732
	8	65377497	65384794
	11	21184338	21192141
	11	26076972	26083126
	1	23693197	23700911
	1	27940280	27948070
	1	36155282	36163063
	5	13342893	13353515
	5	13913510	13921298
	9	24350671	24356003
	9	32963967	32971019
	3	45969396	45977197
	3	49410465	49418235
	4	2087998	2096696
	4	24947560	24957116
	2	62149220	62156782
	6	23352846	23357331

EVEs	Chromosome	Start	End
	6	29906188	29913967
	6	41194741	41202241
	6	47368122	47375735
EgFLOR_2	7	6433537	6441429
	7	38351411	38358410
	4	18262117	18269137
	5	13342905	13352773
	5	13914263	13921298
	8	6849737	6856758
	8	36161233	36168252
	1	23693928	23700892
	3	45969419	45976424
	11	21185113	21192097
	2	62149231	62156238
	6	29906965	29913951
	9	32963967	32971229
EgFLOR_3	5	36445030	36452791
	7	6433805	6441411
EgFLOR_4	7	3126120	3133908
	7	38351411	38358967
	7	41547230	41554960
	7	43159241	43166137
	7	47515372	47522956
	11	21184346	21192141
	11	26076972	26082997
	8	6849737	6857512
	8	65377497	65384786
	4	2087999	2096696
	4	18261363	18269146
	4	24947733	24957116

EVEs	Chromosome	Start	End
	1	23693208	23700901
	1	27940280	27947893
	1	36155282	36163055
	2	62149222	62156782
	5	13913700	13921298
	9	24350679	24356003
	9	32963967	32971019
	3	45969396	45977190
	3	49410465	49418227
	6	29906196	29913967
	6	41194741	41202241
	6	47368122	47375740
