# Supplementary material for: Genome-wide analysis of EgEVE_1, a transcriptionally active endogenous viral element associated to small RNAs in Eucalyptus genomes
Source: Genet Mol Biol. 2017 Feb 23;40(1 Suppl 1):217–25. doi: 10.1590/1678-4685-GMB-2016-0086 (PMC5452135; doi:10.1590/1678-4685-GMB-2016-0086)
Supplement: Supplementary file 3 [file 1415-4757-gmb-1678-4685-GMB-2016-0086-Suppl02.pdf]

Table S2 - Expressed sequence tags (ESTs) matching to EVE elements.

Query source (Complete LTR-RTE families)	EST database	*Eucalyptus* species/NCBI Acc	Matching Query		Identity (%)
			Start	End	
EgEVE1	Sanger ESTs from NCBI	*E. camaldulensis* (FY783514)	2799	3588	83
		E. camaldulensis (FY783581)	2799	3562	83
		E. camaldulensis (FY783608)	2819	3507	84
	Assembly 454 ESTs Eucagen	*E. camaldulensis* (FY808456)	2799	3588	83
**EgFLOR_1**	Eucatool	*E. grandis* (BV682108)	7094	7305	91
