# Supplementary material for: Genome-wide analysis of EgEVE_1, a transcriptionally active endogenous viral element associated to small RNAs in Eucalyptus genomes
Source: Genet Mol Biol. 2017 Feb 23;40(1 Suppl 1):217–25. doi: 10.1590/1678-4685-GMB-2016-0086 (PMC5452135; doi:10.1590/1678-4685-GMB-2016-0086)
Supplement: Supplementary file 4 [file 1415-4757-gmb-1678-4685-GMB-2016-0086-Suppl03.pdf]

Table S3 - sRNAs mapped to *Eucalyptus* EVEs.

Nt size	*EgEVE_1*	*EgFLOR_1*	*EgFLOR_2*	*EgFLOR_3*	*EgFLOR_4*
16nt	7	2	2	2	2
17nt	6	2	2	2	1
18nt	6	0	1	0	1
19nt	4	3	0	1	2
20nt	4	4	1	0	1
21nt	28	10	2	4	2
22nt	23	26	1	4	6
23nt	29	14	2	3	3
24nt	312	116	4	10	47
25nt	14	5	1	1	2
26nt	1	1	0	0	0

nt:nucleotide
